# Results from the ENJOY MAP for HEALTH: a quasi experiment evaluating the impact of age-friendly outdoor exercise equipment to increase older people’s park visitations and physical activity

**DOI:** 10.1186/s12889-024-19042-3

**Published:** 2024-06-22

**Authors:** Pazit Levinger, Bronwyn L. Dreher, Sze-Ee Soh, Briony Dow, Frances Batchelor, Keith D. Hill

**Affiliations:** 1 National Ageing Research Institute, Royal Melbourne Hospital, PO Box 2127, Melbourne, 3050 Australia; 2 Institute for Health and Sport, Victoria University, Melbourne, Australia; 3 Rehabilitation, Ageing and Independent Living (RAIL) Research Centre, Monash University, Melbourne, Australia; 4 School of Primary and Allied Health Care, Monash University, Melbourne, Australia; 5 Faculty of Medicine, Dentistry and Health Sciences, University of Melbourne, Melbourne, Australia; 6 School of Nursing and Midwifery, Deakin University, Waurn Ponds, Australia; 7 Department of Physiotherapy, The University of Melbourne, Melbourne, Australia

**Keywords:** Seniors Exercise Park, Physical activity, Parks, Older people, Built environment

## Abstract

**Background:**

Recreational parks can play a significant role in older people’s health, with emerging evidence suggesting that changes in the physical environment, such as refurbishments of local parks, can increase park visitations and physical activity engagement. The ENJOY MAP for HEALTH aimed to evaluate the impact of Seniors Exercise Park installations and associated capacity building activities on older people’s park visitation, and park-based physical activity.

**Method:**

The ENJOY MAP for HEALTH was a quasi-experiment study design that involved the installation of specialised Seniors Exercise Park equipment as part of park refurbishment, supported by promotion and community capacity building activities in six municipalities in Victoria, Australia. Direct observations of park users took place prior to park upgrades, one-month post upgrade and 12-months from baseline. The overall number and characteristics of park visitors, and the type and level of physical activity undertaken, were summarised descriptively. Generalised linear models were used to examine the impact of park refurbishment (equipment installation and site activation) on the total number of older people observed in the park, and their engagement in physical activity, accounting for site and seasonal effects.

**Results:**

Overall number of visits increased following park upgrades, with the largest number of visitors observed one-month post upgrade (*n* = 12,501). The proportion of older people observed at the parks remained relatively low prior to and one-month post upgrade compared to other age groups. However, after adjusting for site and seasonal effects, the number of older people observed in the parks increased significantly post upgrade and site activation compared to prior to the refurbishment (incidence rate ratios (IRR) 3.55; 95% CI 2.68, 4.70). The number of older people observed to be exercising at the Seniors Exercise Park also increased by 100% at 12-months post-installation relative to one-month post upgrade (IRR 2.00; 95% CI 1.26, 3.17).

**Conclusion:**

Installation of the Seniors Exercise Parks and the supportive programs and activities following six park upgrades resulted in an increase in older people’s park visitation and engagement in physical activity. Community engagement and training of volunteers with the support of local governments are likely to contribute to the increased park usage by older people.

**Trial registration:**

This trial was registered with the Australian New Zealand Clinical Trials Registry. Trial registration number ACTRN12621000965808. https://www.anzctr.org.au/Trial/Registration/TrialReview.aspx?id=380745&isReview=true.

## Introduction

Physical inactivity is a major public health concern especially in older age. Older adults in particular have lower levels of physical activity with a small proportion (13-41.8%) meeting the recommended physical activity guidelines [1, 2]. With the growing ageing population, engagement in regular physical activity is essential for maintaining good health and preventing chronic diseases among older people [3–5]. Physical activity in the outdoor environment, such as in local parks, offers many health benefits, including physical, mental and social benefits [6–8]. The usage of local parks as a place to engage in planned and incidental activities has been recognised as a valuable mode to maintain physical health [9], with increased benefits for older people [10, 11]. In recent years, the availability of outdoor exercise equipment in local parks has become quite popular as an active space to exercise for outdoor leisure [12–14]. The usage of outdoor exercise equipment for older people has also shown various health benefits [15, 16] with emerging evidence for the need to create well designed active spaces with age-suitable exercise equipment [17–19].

Changes in the physical environment, such as refurbishments of local parks, have shown positive impacts on park visitations and engagement in physical activity [20]. Research interventions examining the impact of park improvements/environmental change, incorporating natural experimental design, are commonly used to examine causal associations between the built environment and physical activity [21, 22]. A large study conducted in Australia has demonstrated that upgrades of local parks, including the installation of various children’s play-spaces, increased park visitation and encouraged visitors to be physically active [22]. However, the latter study was focused on installation and upgrade of children’s play equipment; with limited studies evaluating the impact of age-suitable outdoor exercise equipment on older people’s park visitation and engagement in physical activity.

With the growing number of the older demographic and the need to provide opportunities for everyone to engage in physical activity in public spaces, the number of age-suitable exercise equipment has increased in recent years. In the past several years our work has involved investigating the usage and benefits of a specialised outdoor exercise equipment, the Seniors Exercise Park, on older people’s health. The Seniors Exercise Park integrates multimodal exercise stations that target balance (unstable/uneven surfaces), strength, flexibility and functional movements, which can all positively contribute to improve the physical function and independence of older people. Our research has demonstrated various health benefits of the Seniors Exercise Parks [23–26], highlighting their potential impact as an important public health infrastructure investment in promoting physical activity for older people [18, 27].

Engagement with local governments and the community for wider implementation of initiatives to include more specialised equipment in the community can potentially have greater public health benefits. However, unlike installing play equipment for children, which naturally results in spontaneous play and increased physical activity [22], older people may require a systematic implementation and usage plan to maximise uptake and ensure safe use of installed equipment. Therefore, effective communication, strategic planning and community capacity building activities are important to consider to complement park upgrade/refurbishment [18]. The present study, the ENJOY MAP for HEALTH (Exercise interveNtion outdoor proJect in the cOmmunitY for older people - More Active People for HEALTHier communities), is built on our previous work to improve the built environment to promote physical activity for older people. The ENJOY MAP for HEALTH is a quasi-experiment study that aimed to evaluate the impact of the Seniors Exercise Park installation and associated capacity building activities on older people’s park visitation and park-based physical activity.

## Method

**Fig. 1:**
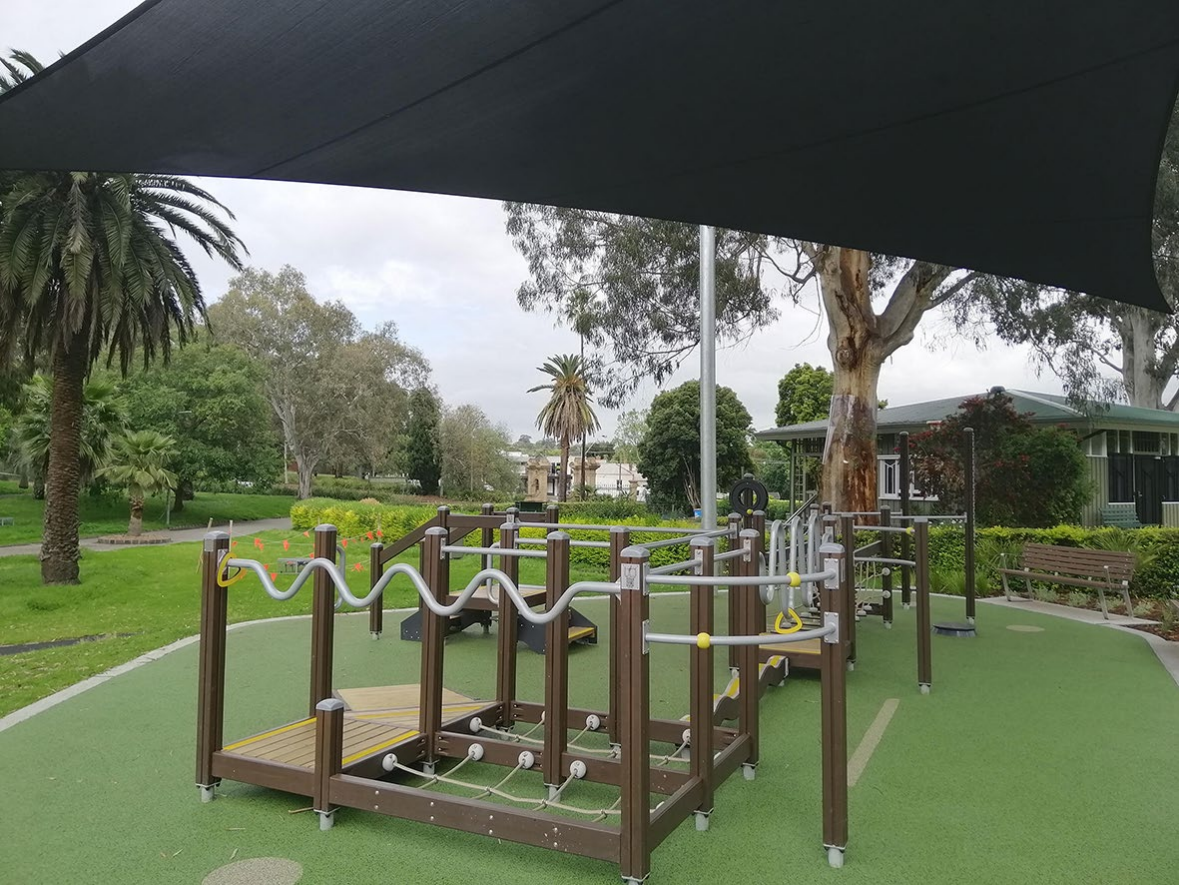
The Seniors Exercise Park at Victoria Park, Kew, Melbourne

The ENJOY MAP for HEALTH was a quasi-experimental pre-post study design that involved the installation of the specialised Seniors Exercise Park equipment (Fig. 1) supported by promotion and community capacity building activities in six municipalities in Victoria, Australia. The project included four stages in each of the six participating sites with staggered commencement of two sites per block including: site construction and development, promotion and marketing, capacity building and training, evaluation and sustainability. Further details have been provided in the study protocol [28].

### Study setting and partners

The following councils (local governments) participated in the study: Knox City Council (Carrington Park, Knoxfield), Banyule City Council (Ivanhoe Park, Ivanhoe), City of Ballarat (Mt Pleasant Reserve, Mt Pleasant), City of Frankston (Wingham Park, Frankston), City of Boroondara (Victoria Park, Kew), and Bayside City Council (Thomas St Reserve, Hampton). The project was implemented in staggered stages (in three blocks, two local governments per block) where each local government underwent site refurbishment in a specific timeline based on the local government’s site upgrade plan. Park refurbishments included the installation of the Seniors Exercise Park, other areas of the parks (children’s play spaces) and other amenities (e.g., water fountain, shaded area, benches). There was variation between the six parks in overall site size, surrounding areas and amenities, and the additional play equipment available.

Evaluation of the impact of site refurbishment and equipment installation on park visitation and physical activity engagement of older people was conducted using observations of park users, and the collection of reports/audits of programs offered by the participating local government partners and/ or the respective local health/leisure providers. All procedures were conducted in compliance with the National Statement on Ethical Human Research and the Australian Code for the Responsible Conduct of Research. Ethical approval was obtained from Monash University Human Research Ethics Committee, Melbourne Australia (Project ID: 25499). The study was designed according to the strengthening the reporting of observational studies in epidemiology (STROBE) statement [29].

### Study population

All park visitors were included in the observation data collection. Consent from park visitors was not required as participants remained anonymous and the behaviours occurred in a public setting where there was no breach of privacy (approved by Monash University Human Research Ethics Committee, Melbourne Australia (Project ID: 25499)). No personal or identifiable data was collected during the observation period.

### Assessments

#### Outcome measures

##### Park observation and Seniors Exercise Park equipment usage

Observation of park and Seniors Exercise Parks visitation and usage took place prior to site construction (baseline, T0) and at two time points: one-month after the site was open for public (T1) and 12-months after baseline (T2). The 12-months evaluation was planned to take place at the same time of the year as the baseline assessments to account for potential seasonal effects.

Detailed information about the observation method (the System for Observing Play and Recreation in Communities (SOPARC)) is provided in the study protocol [28]. In brief, the SOPARC is a reliable and feasible instrument based on momentary time sampling techniques in which systematic and periodic scans of individuals and contextual factors within pre-determined target areas in parks are made [30]. The direct observation collected information on community park use, characteristics of park visitors, activities at the parks, and estimated age groups (child-infant up to 12 years, teen − 13–20 years, adult – 21–59 years, older people – 60 + years of age). Breakdown of actual activity type included walking, jogging/running, cycling, strength, fitness station, sports game (ball game e.g., soccer), playground (any activity using play space equipment), other play (chasing, playing with ball), or inactivity (standing, sitting, lying down, chess/card games, reading, eating).

As this study focused on the usage of the Seniors Exercise Park, additional data was collected in the area of the Seniors Exercise Park at the follow up data collection periods (one-month post and 12 month post), including interaction with the outdoor exercise equipment (i.e., ‘using equipment as intended’ or ‘playing/looking/sitting’ on the equipment) [31]. Each site was divided into targeted areas and systematic scans were conducted over a 7-day period (Monday-Sunday) with a total of 11 scans per day as follows (22 and 55 scans for weekend and weekdays respectively): every 30 min of all park visitors in the study area during early morning (07:00–08:30), mid-day (11:30–13:00) and late afternoon (16:00–18:30) [32]. Research staff were trained on how to use the SOPARC using the user guide and available resources, in addition to a practical on-site session at one of the parks. Staff attended at least one familiarisation session at each park prior to conducting the observation data collection relevant to that park. The practical session was run over several recording periods until staff were consistent with the data being collected.

### Review and audit of programs/activities at the park

Information about programs/activities (such as session frequency, method of session delivery, attendees number) provided by the local governments or other organisations was collected from local government staff (from the Positive Ageing team or equivalent).

### Park audits

Information about park characteristics (e.g., size), features and amenities before and after park upgrade was collected from each local government’s open space team including any additional equipment/features/amenities, play space equipment, play space areas fitness equipment, seated areas/benches, water fountain, roofed shade area and more (see Table 1).

**Table 1:** Parks amenities and features post refurbishment at the six locations

Amenities /features	Ivanhoe Park	Mt Pleasant Reserve	Thomas St Reserve	Carrington Park	Victoria Park	Wingham Park
**Size**	52,827m^2^	27,000 m^2^	38,143.67 m^2^	75,576 m^2^	6,000 m^2^	28,759.6 m^2^
**Sport play court/field (e.g. basketball/football/netball)**	Basketball/netball half court Oval	Basketball half court Oval	-	√ Radio controlled car race track Oval	-	-
**Water fountain/ drinking bubbler**	√	√	√	√	√	√
**Public toilet**	√	√	√	-	√	-
**Picnic/BBQ area**	√	√	√ additional/upgrade	√	√	√
**Seated benches**	√ additional bench seats	√ additional bench seats	√ additional bench seats	√ additional bench seats	√ additional bench seats	√ additional bench seats
**Shade-sail**	-	-	√ x3 areas	√ x 1 area	√ x3 areas	-
**Roofed shade area**	√ 1 timber shelter	√ x 1 timber shelter	√ x 5 timber shelters	√ x concrete area in near by facility	√ x 1 timber shelter	√ 1 timber shelter
**Kids playground/play-space**	√ Additional of play space equipment and structure	Climbing frame, slide, swing set, rocker, spinner and ropes/balancing apparatus.	√ Additional of play space equipment and structure	√ Additional of play space equipment and structure	√ Additional of play space equipment and structure	√
**Sand pit**	-	-	√	-	√	-
**Water station/play area**	-	-	√	-	√	
**Skate/scooter/ride track**	-	-		-	√	
**Exercise equipment**	√ Seniors Exercise Park	√ Seniors Exercise Park	√ Seniors Exercise Park	√ Seniors Exercise Park	√ Seniors Exercise Park Fitness stations	√ Seniors Exercise Park Gym-based machines
**Table and seats throughout**	√	√	√	√	√	√
**Walking track**	√ circuit paths	√ circuit paths	√ circuit paths	√ circuit paths	√ perimeter of park and within areas	√ circuit paths
**Garden beds**	-	-	√	√	√	-
**Tree planting**	√	√	√	√	√	√
**Natural landscaping**	√	-	**√**	√	√	√
**Dog area (off leash/fenced area)**	-	-	√ off leash	√ off leash	√ off leash	-
**Buildings/facility centers near by**				Senior Citizens Centre		Neighbourhood Community Centre
**Site participation timeline (inclusive of construction and data collection periods)**	Sep 2020*-Oct 2021	Sep 2020*-Oct 2021	Oct 2020*-June 2022	Sep 2021*- Oct 2022	Jan 2022*-June 2023	Aug 2022- Sep 2023

*Note* Amenities/park features that have been upgraded or added as part of parks refurbishment are marked with grey

*Interruption due to COVID19: travel/access restrictions/delay in construction completion

### Intervention - Seniors Exercise Park Installation and Site Activation

#### The Seniors Exercise Park Equipment Installation

The Seniors Exercise Park equipment comprises multiple equipment stations that target specific function or movement, static and dynamic balance, and functional movement (e.g., walking up/down stairs, sit to stand) designed specifically for older people (Fig. 1). Installation of the outdoor equipment was standardised across all sites where local governments were provided with guidance around suitable flooring/surface (rubber surface or equivalent) and other safety measures for installation [19, 33]. Instructional signage with illustrations and information on how to use the equipment was also installed, as well as seated benches for resting within the area. The usage of the equipment has been reported to be safe for older people (aged 60 years and over and including those with increased risk of falls) and those living with dementia, with no serious adverse events [23, 26, 34].

#### Site activation

The ENJOY MAP for HEALTH included several strategies embedded to ‘activate a site’ to enable positive effects on physical activity behavior and sustainability, including: (1) promotion and marketing (led by the councils’ marketing and promotion team, or equivalent) and (2) capacity building (training of volunteers and allied health professionals) led by the research staff. In addition, all exercise equipment stations at each park were fitted with small metal plates that incorporated Quick Response (QR) codes linked to an online progressive web application. The web application content was developed by the research staff in consultation with older people and incorporated specific exercise instructions, videos, and safety tips. Visitors were able to scan the QR code with their mobile phone.

The rationale of including the activation strategies was to increase reach in the community, enable safe usage of the Seniors Exercise Park by visitors, increase knowledge and community upskilling, and increase visitation to the park.

### 1) Promotion and marketing

Ongoing communication and promotion (led by the councils’ communication and marketing team in consultation with the research staff) involved the design of promotional material to be distributed in online and offline channels such as digital (Facebook, Instagram, website) and physical promotion (banner, posters, flyers) platforms. Community events (official park launch, come-and-try sessions) were also organised by the local governments to engage and reach community members.

### 2) Capacity building - upskilling and training– knowledge transfer

Community capacity building activities included (a) training modules, and (b) community health care engagement.

### 2a) Training modules - Allied Health professionals training workshops and community volunteers (train the trainer)

#### Allied Health Professionals training

Allied Health Professionals training included a half day training workshop delivered at each site for local allied health professionals (e.g., physiotherapists, exercise instructors, accredited exercise physiologists). The training covered both theoretical and practical components, including safe Seniors Exercise Park use, exercise prescription and program design, and risk management. The number of registrations for training was collected. The training workshops were delivered by the research staff, who were accredited exercise physiologists and/or physiotherapists.

#### Train the trainer module

A five-week twice weekly training module was designed and delivered to community volunteers (ENJOY champions) that included nine practical training sessions using the Seniors Exercise Park (1.5 h duration incorporating interactive teaching with hands-on demonstrations) and a final theoretical session for 3 h incorporating risk management, physical activity and healthy lifestyle tips. The volunteers were recruited by the councils’ Positive Ageing team (or equivalent) and the training module was delivered by the research staff (who were allied health professionals: physiotherapists and/or accredited exercise physiologists). This module aimed to upskill volunteers to enable them to support utilisation of the Seniors Exercise Park by their peers and more widely in the local community, in order to maximise older people’s community engagement and physical activity participation. Adherence was monitored using a participation log.

### 2b) Community health care and leisure centre providers engagement

The research team worked closely with the Positive Ageing and Disability and Community Development teams (or equivalent) at each local government to identify and develop relationships with local health care/leisure providers. Engagement throughout the various project stages included involvement in training, project meetings, and community events.

### A targeted outcome for ENJOY MAP for HEALTH project

An intervention that incorporates change in the physical built environment with other community engagement is likely to result in substantial increase in physical activity participation [35, 36]. We hypothesised that the intervention (Seniors Exercise Park installation and site activation) would result in *at least* a twofold increase (100%) in the number of visitors (older people) between baseline to the 12-months follow up.

### Statistical analysis

Descriptive statistics (counts and relative proportion) were used to report the overall numbers of park visitors, visitor characteristics, and the activity types of visitors at the different time points. Descriptive statistics were also used to report the usage of the Seniors Exercise Park by the different age groups and the type of activities undertaken in each area.

Generalised linear models [37] were used to examine the impact of park refurbishment (equipment installation and site activation) on the total number of older people observed in the park, and the number of people walking and being physically active in the park. Specific terms were included in the model for the intervention effect, the site effects and seasonal effects. Interactions between the intervention effect (i.e. park refurbishment) and seasonal effects were also examined. Overdispersion was handled using a negative binomial distribution, and model effects were reported as incidence rate ratios (IRR) and 95% confidence intervals (CIs) given that the outcome was count data (i.e. the number of older visitors). Univariate negative binomial regression models were also used to compare the number of older people using the Seniors Exercise Park following installation. All statistical analyses were conducted using Microsoft Excel and Stata/SE18.0 (StataCorp College Station, Texas, USA).

## Results

The project was executed sequentially based on each local government’s timelines and planning around construction work. Data collection took place between September 2020 and September 2023, with park refurbishments occurring at various times between October 2020 and October 2022. As the project was conducted during the COVID-19 lockdown periods, travel and access restrictions impacted some data collection timelines and delayed site construction completion due to shortage of material or staff (details in Table 1). The variations in construction time length (1–8 months) further impacted the intervention period exposure at each site to 6–10 months. Following the intervention activities, as a natural consequence of intervention impact, it was observed that programs/sessions were run outside the observation scan periods. As a result, three additional scans (9:30-10am, 10–10:30am, 10:30-11am) were added to the Seniors Exercise Park areas to enable systematic capturing of activities outside the original scan periods (T2 with additional scans). Sensitivity analyses were therefore performed using data obtained from these additional scans to determine whether the impact of park refurbishment on the number of older people observed in the park changed.

Details about park features, sizes and amenities post refurbishment are provided in Table 1. The six parks varied in the additional features/amenities that were added/upgraded as part of park refurbishment; the changes included the addition of several playspaces and play equipment for children, passive areas (e.g., benches, barbeques) and adult exercise equipment (Seniors Exercise Park, gym-machine equipment).

### Training, promotion and activities/sessions at the parks

A total of six workshop training sessions were delivered to allied health professionals with a total of 128 attendees. Attendees’ occupations included: 61 (47.6%) physiotherapists/exercise physiologists/occupational therapists; 14 (10.9%) fitness instructors, 24 (18.7%) allied health assistants, 12 (9.4%) other health related occupations (nurses/dietician/psychologist/chiropractors), 10 (7.8%) council staff (e.g., inclusion officer) and 7 (5.5%) others (e.g., volunteers). Six training modules were delivered for community volunteers (champions), one at each location, with a total of 53 participants (20 (37.7%) men and 33 (62.3%) women) with an average of 80% attendance across the 10 sessions. The majority of volunteers (94.3%) were 60 years of age and over. These champions were formally registered as volunteers with their respective local government. Following the training of the champions, all participating local governments scheduled free weekly community sessions (come and try/drop in sessions) guided by the champions between 1 and 2 times per week. At the completion of the study five out of the six sites maintained the community sessions regularly. In one local government, due to COVID-19 lockdown and associated interruption, these sessions were stopped and were not resumed. Each local government organised an official launch (opening of the park) following park upgrades, which was promoted through social media, local government’s website or newsletter. In three of the sites, the local community centres practitioners reported to be using the Seniors Exercise Park as part of their physical activity programs for older people.

### Park usage

An increase in the number of visitors was observed for the two timepoints following park upgrades, with the largest number of visitors observed one-month post upgrade (T1, *n* = 12,501). The proportion of older people observed at the parks ranged between 4.5 and 8.3% (170–509) and remained relatively low prior to and one-month post park upgrades, Table 2. An increase in the number of older visitors was demonstrated at the 12-months follow up (T2) especially in the observation that included additional scans. At baseline 52.4% (*n* = 89 out of 170, T0) of older people were observed to be physically active, with walking being the most common form of physical activity (50.5%, *n* = 86 out of 170). The proportion of older people who engaged in physical activity increased by 2.8% (*n* = 191, 55.2%) and 11.1% from baseline to 12 month follow up (T2, *n* = 277, 63.5%), with the latter taking into consideration the additional scans. In addition to walking, it was observed that older people were also using the outdoor exercise equipment at one-month (T1) and 12 months after baseline (T2). This was more noticeable with the additional scans: 30.3% (*n* = 132) of older people were observed exercising using outdoor exercise equipment, and only 23.8% (*n* = 104) observed walking.

**Table 2:** Park visitors’ demographic and activities in the various timepoints before and after parks upgrade

				Observed activity									
**Estimated age group n (%)**	**Total visitors**	**Female**	**Male**	**Walking**	**Jog/run**	**Cycling**	**Strength**	**Fitness station**	**Sport game**	**Playground**	**Other play**	**Physically active**	**Inactive**
**Baseline (T0)**													
All age groups	3770	2102 (55.7)	1668 (44.3)	878 (23.2)	59 (1.5)	92 (2.4)	16 (0.4)	1 (0.1)	201 (5.3)	452 (11.9)	93 (2.4)	1792 (47.5)	1978 (52.4)
Child (1–12 year)	1788 (47.4)	969 (55.7)	819 (44.2)	227 (12.7)	33 (1.8)	58 (3.2)	0	0	76 (4.2)	383 (21.4)	91 (5.0)	868 (48.5)	920 (51.4)
Teen (13–20 year)	219 (5.8)	113 (54.2)	106 (45.8)	44 (20.1)	7 (3.2)	17 (7.7)	0	0	45 (20.5)	28 (12.7)	0	141 (64.4)	78 (35.6)
Adult (21–59 year)	1593 (42.2)	928 (51.6)	665 (48.4)	521 (32.7)	18 (1.1)	16 (1.0)	15 (0.9)	1 (0.1)	80 (5.0)	41 (12.7)	2 (0.1)	694 (43.6)	899 (56.4)
Older people (60+)	170 (4.5)	92 (58.2)	78 (41.7)	86 (50.5)	1 (0.5)	1 (0.5)	1 (0.5)	0	0	0	0	89 (52.4)	81 (47.6)
**One-month post upgrade (T1)**													
All age groups	12,501	6959	5542	1232 (9.9)	156 (1.2)	201 (1.6)	20 (0.2)	145 (1.2)	62 (0.5)	4129 (33.0)	826 (6.6)	6771 (54.2)	5730 (45.8)
Child (1–12 year)	6941 (55.5)	3660 (52.7)	3281 (47.3)	400 (5.8)	122 (1.8)	179 (2.6)	3 (0.04)	56 (0.8)	12 (0.2)	3823 (55.1)	776 (11.2)	5371 (77.4)	1570 (22.6)
Teen (13–20 year)	477 (3.8)	179 (37.5)	298 (62.5)	44 (9.2)	11 (2.3)	11 (2.3)	0 (0)	16 (3.3)	22 (4.6)	126 (26.4)	18 (3.8)	248 (52.0)	229 (48.0)
Adult (21–59 year)	4574 (36.6)	2812 (61.5)	1762 (38.5)	653 (14.3)	23 (0.5)	11 (0.2)	15 (0.3)	36 (0.8)	28 (0.6)	163 (3.6)	30 (0.7)	959 (21.0)	3615 (79.0)
Older people (60+)	509 (4.1)	308 (60.5)	201 (39.5)	135 (26.5)	0 (0)	0 (0)	2 (0.4)	37 (7.3)	0 (0)	17 (3.3)	2 (0.4)	193 (37.9)	316 (62.1)
**12 months from baseline (T2)**													
All age groups	5190	2836 (54.6)	2354 (45.4)	831 (16.0)	135 (2.6)	70 (1.3)	4 (0.1)	97 (1.9)	88 (1.7)	1798 (34.6)	209 (4.0)	3232 (62.3)	1958 (37.7)
Child (1–12 year)	2540 (48.9)	1320 (52.0)	1220 (48.0)	193 (7.6)	84 (3.3)	60 (2.4)	0	15 (0.6)	48 (1.9)	1547 (60.9)	173 (6.8)	2120 (83.5)	420 (16.5)
Teen (13–20 year)	223 (4.3)	99 (44.4)	124 (55.6)	35 (15.7)	27 (12.1)	8 (3.6)	1 (0.4)	2 (0.9)	23 (10.3)	62 (27.8)	10 (4.5)	168 (75.3)	55 (24.7)
Adult (21–59 year)	2081 (40.1)	1224 (58.8)	857 (41.2)	499 (24.0)	22 (1.0)	2 (0.1)	2 (0.1)	25 (1.2)	17 (0.8)	162 (7.9)	24 (1.1)	753 (36.2)	1328 (63.8)
Older people (60+)	346 (6.7)	193 (55.8)	153 (44.2)	104 (30.1)	2 (0.6)	0	1 (0.3)	55 (15.9)	0	27 (7.8)	2 (0.6)	191 (55.2)	155 (44.8)
**T2 with additional scans**													
All age groups	5293	2908 (54.9)	2385 (45.1)	831 (15.7)	135 (2.5)	70 (1.3)	22 (0.4)	175 (3.3)	88 (1.7)	1798 (34.0)	209 (3.9)	3328 (62.9)	1965 (37.1)
Child (1–12 year)	2540 (48.0)	1320 (52.0)	1220 (48.1)	193 (7.6)	84 (3.3)	60 (2.4)	0	15 (0.6)	48 (1.9)	1547 (60.9)	173(6.8)	2120 (83.5)	420 (16.5)
Teen (13–20 year)	223 (4.2)	99 (44.4)	124 (55.6)	35 (15.7)	27 (12.1)	8 (3.6)	1 (0.4)	2 (0.9)	23 (10.3)	62 (27.8)	10 (4.5)	168 (75.3)	55 (24.7)
Adult (21–59 year)	2094 (39.6)	1236 (59.0)	858 (41.0)	499 (23.8)	22 (1.1)	2 (0.1)	11 (0.5)	26 (1.2)	17 (0.8)	162 (7.7)	24 (1.1)	763 (36.4)	1331 (63.5)
Older people (60+)	436 (8.2)	253 (58.1)	183 (42.0)	104 (23.8)	2 (0.4)	0	10 (2.3)	132 (30.3)	0	27 (6.2)	2 (0.4)	277 (63.5)	159 (36.5)

### Impact of park refurbishment on number of older people in the park

After adjusting for site and seasonal effects, the number of older people in the park increased significantly post upgrade compared to baseline (IRR 3.55; 95% CI 2.68, 4.70). The number of older visitors remained 91% higher 12-months post installation relative to prior to the upgrade (IRR 1.91; 95% CI 1.18, 3.08), but this was 46% less compared to one-month post park upgrade (IRR 3.55; 95% CI 2.68–4.70). Similarly, the number of older people who were physically active increased by 96% one-month after upgrade (IRR 1.96; 95% CI 1.40, 2.73) and 83% 12-months after baseline (IRR 1.83; 95% CI 1.12, 3.00) after controlling for other covariates. Similar significant results were observed using data from the additional scans except that the size of the effect was greater, where we observed a 270% increase in the number of older people (IRR 3.70; 95% CI 2.72, 5.05) compared to 255% using the data from the original scans (Table 3). No statistically significant interactions were observed between park refurbishment (before and after) and season (winter, spring, summer and autumn).

**Table 3:** Impact of park refurbishment on the number of older people*

	Original data		Includes data from additional scans	
	Total number	Physically active	Total number	Physically active
Timepoint				
Baseline	1.0 (reference)	1.0 (reference)	1.0 (reference)	1.0 (reference)
Post-installation	**3.55 (2.68, 4.70)**	**1.96 (1.40, 2.73)**	**3.70 (2.72, 5.05)**	**2.55 (1.63, 3.99)**
12-months	**1.91 (1.18, 3.08)**	**1.83 (1.12, 3.00)**	**2.23 (1.35, 3.70)**	**2.46 (1.61, 3.77)**

*Models adjusted for site and seasonal effects

All data reported as incidence rate ratios (IRR), 95% CI

Bolded values *p ≤* 0.05

#### Seniors Exercise Park area usage

The proportion of older people observed at the Seniors Exercise Park areas increased following the installation from 6.9% (*n* = 76 out of 1101 visitors, T1) at one-month post installation to 16.6% (*n* = 73 out of 440 visitors, T2) and 29.3% (*n* = 154 out of 525 visitors, T2) at the 12-months from baseline (following site activation), with a third of all older park visitors observed at those areas, Table 4. Relative to one-month post-installation, the number of older people observed to be exercising at the Seniors Exercise Park increased significantly after 12-months (IRR 2.00; 95% CI 1.26, 3.17) although there was no difference observed in the number of older people at the Seniors Exercise Park areas (IRR 0.96; 95% CI 0.70, 1.32). Sensitivity analyses using data from the additional scans indicated, however, that the number of older people observed at the Seniors Exercise Park areas increased 12-months post-installation compared to one-month post upgrade (IRR 2.02; 95% CI 1.54; 2.67) (Table 5). A small proportion of older people were observed to be inactive (looking at or siting in the area) following the intervention (26% and 14.9% at T2, and T2 with additional scans respectively) with no older visitors passing by without using it, Table 4.

**Table 4:** Seniors Exercise Parks’ visitors and usage demographics after parks upgrade

		Proportion relative to the counts within the Seniors Exercise Park areas						
**Estimated Age group n (%)**	*Proportion relative to overall park counts	**Total visitors** **n (%)**	**Female**	**Male**	**Exercise**	**Play**	**Look/sit**	**Pass by**
**One-month post upgrade (T1)**								
All age groups	1101 (9.7)	1101	632 (57.4)	469 (42.6)	79 (7.2)	491 (44.6)	516 (46.9)	15 (1.4)
Child (1–12 year)	562 (8.8)	562 (51.1)	312 (55.5)	250 (44.5)	31 (5.5)	460 (81.9)	69 (12.3)	2 (0.4)
Teen (13–20 year)	51 (12.0)	51 (4.6)	15 (29.4)	36 (70.6)	4 (7.8)	11 (21.6)	31 (60.8)	5 (9.8)
Adult (21–59 year)	412 (9.9)	412 (37.4)	254 (61.7)	158 (38.3)	17 (4.1)	17 (4.1)	373 (90.5)	5 (1.2)
Older people (60+)	76 (17.6)	76 (6.9)	51 (67.1)	25 (32.9)	27 (35.5)	3 (3.9)	43 (56.6)	3 (3.9)
**12 months from baseline (T2)**								
All age groups	440 (8.5)	440	258 (58.6)	182 (41.4)	79 (17.9)	220 (50.0)	132 (30.0)	9 (2.1)
Child (1–12 year)	213 (8.4)	213 (48.5)	121 (56.8)	92 (43.2)	3 (1.4)	197 (92.5)	11 (5.2)	2 (0.9)
Teen (13–20 year)	17 (7.6)	17 (3.8)	8 (47.1))	9 (52.9)	2 (11.8)	14 (82.4)	1 (5.9)	0
Adult (21–59 year)	137 (6.6)	137 (31.1)	80 (58.3)	57 (41.6)	20 (14.6)	9 (6.6)	101 (73.7)	7 (5.1)
Older people (60+)	73 (21.1)	73 (16.6)	49 (67.1)	24 (32.9)	54 (74.0)	0	19 (26.0)	0
**T2 with additional scans**								
All age groups	525 (9.9)	525	316 (60.2)	209 (39.8)	157 (29.9)	220 (41.9)	139 (26.5)	9 (1.7)
Child (1–12 year)	213 (8.4)	213 (40.6)	121 (56.8)	92 (43.2)	3 (1.4)	197 (92.5)	11 (5.2)	2 (0.9)
Teen (13–20 year)	17 (7.6)	17 (3.2)	8 (47.1)	9 (52.9)	2 (11.8)	14 (82.4)	1 (5.9)	0
Adult (21–59 year)	141 (6.7)	141 (26.9)	83 (58.9)	58 (41.1)	21 (14.9)	9 (6.4)	104 (73.6)	7 (5.0)
Older people (60+)	154 (35.3)	154 (29.3)	104 (67.5)	50 (32.5)	131 (85.1)	0	23 (14.9)	0

*proportion relative to the same age group

**Table 5:** Number of older people using Seniors Exercise Parks post installation

	Total number of visitors	Exercising/physically active
Timepoint		
Post-installation	1.0 (reference)	1.0 (reference)
12-months post-installation	0.96 (0.70, 1.32)	**2.00 (1.26, 3.17)**
Additional scans	**2.02 (1.54, 2.67)**	**4.85 (3.21, 7.34)**

All data reported as unadjusted incidence rate ratios (IRR) and 95% CI

Bolded values *p ≤* 0.05

## Discussion

Local parks are known as valuable places for people to engage in leisure and physical activities with many health benefits reported for older people. However, older people make up the lowest proportion of local park visitors by age group [10, 22, 38]. Therefore, innovative approaches to increase park visitation and park-based physical activity for this demographic are warranted. To our knowledge this is the first quasi-experimental study that has investigated the impact of park refurbishment including the installation of age-friendly outdoor exercise equipment, complemented by activation activities, on older people’s park visitation and their engagement in physical activity. Importantly, this study used an evidence based outdoor exercise equipment set previously shown to improve health outcomes for older people in the community [16, 24, 39], with targeted promotion and activities to engage communities and local governments (site activation). The present study demonstrated a significant increase in the number of older visitors in the park following both refurbishment and site activation with a greater proportion of older visitors following site activation (at 12-months) compared to baseline. The proportion of older people engaged in physical activity increased by 11% from baseline to follow up when accounting for the additional observation. The study outcomes highlight the importance of including specialised outdoor exercise equipment installation as part of park refurbishment to increase older people’s visitation to local parks and engagement in park-based physical activity, as well as site activation activities.

Previous natural experiment studies evaluating the impact of park refurbishment on park visitation and engagement in physical activity reported mixed results. Upgrade of children’s playspaces resulted in 33% increase in park visitors, mainly children and adults but with no significant increase in older visitors [22]. When examining the impact of outdoor fitness equipment installation, one study reported no significant increase in total visitors or park-based physical activity [40] while another study reported significant increase in engagement in physical activity for all visitors and also older people [41]. The latter study provided induction of several exercise sessions led by accredited exercise professionals, with targeted promotion and marketing to specifically engage older people in using the outdoor gym [41, 42]. Similarly, a study from Brazil reported greater engagement of physical activity in parks with free physical activity classes compared to parks without classes [36]. The present study incorporated several activities targeted to increase older people’s engagement in physical activity, including local promotions and regular free sessions led by volunteers, as well as QR codes and instructional signage. The type of physical activity older people engaged in at the parks following site activation included a wider range of activities than just walking, as observed at baseline. While walking is the most common type of physical activity for older people in parks [43, 44], providing other options for physical activity using different exercise equipment can offer added health benefits [45]. Consequently, the inclusion of supportive organised programs using the equipment seems to facilitate greater visitations to the park, engagement in physical activity and usage of the equipment.

The proportion of older people accessing the Seniors Exercise Park areas relative to the other areas of the parks increased from 17.6% one-month following the installation to 21.1% at 12-months following site activation, with greater visitor numbers observed with the additional morning observation (35.3%). The usage of outdoor exercise equipment (mainly gym-like machine equipment) by older people has been reported to be less than 7% [31, 40]. In the present study, the number of older people exercising using the Seniors Exercise Park one-month following installation (*n* = 27, 35.5%) was demonstrated to be higher compared to previous studies [31, 40]. This was almost doubled (*n* = 54, 74%) following site activation. With the additional morning scans, the number of older people further increased to 85.1% (*n* = 131) which was mainly attributed to the availability of sessions/programs at the Seniors Exercise Park areas.

The largest number of visitors observed was one-month after the refurbished areas were open for public use. There may be several reasons for this. Firstly, the period where data collection occurred at some parks was during COVID-19, when access restrictions were lifted. At this time it was reported globally that general access to public spaces and local parks increased [46, 47]. Secondly, it is expected that a large increase in visitation would occur immediately after major park upgrade, with visitation numbers expected to settle several months later [22]. Interestingly, the proportion of older visitors didn’t increase at the one-month post upgrade and remained similar to baseline. The increased proportion of older visitors occurred mainly at the 12-month follow-up which suggests that the targeted programming and marketing may have had a positive impact on park visitation by older people. This is further supported by the increased proportion of older people observed at the Seniors Exercise Park areas following site activation.

One of the key strategies of the present study for sustainable impact was the capacity building activities provided to the community, local health care providers and local government staff. Training older volunteers and community members enabled knowledge to be shared and maintained locally. This approach is important for the promotion, uptake and maintenance of physical activity [48]. The provision of organised sessions led by older volunteers at the parks was likely to facilitate social interaction and enjoyment, which are both key motivators for older people to participate in physical activity [49]. Given that older people may prefer to exercise with age-matched groups and with people with similar physical appearance and conditions, providing sessions led by older volunteers can further facilitate adherence and social support [50, 51]. Importantly, providing such sessions by volunteers, free of charge to community members, required commitment from the participating local governments in managing and coordinating these activities. Hence, the support and ongoing commitment of local governments are essential to facilitate sustainable impact on older people’s engagement in physical activity in recreational parks to improve their health [18].

This study has several limitations. Firstly, this project did not have control parks as a comparison which may limit the interpretation around the causal impact of the intervention. However, identifying suitable comparable parks in terms of sizes and features would be challenging and perhaps impractical [52]. In addition, given the nature of the intervention and the activities/services provided to ‘activate’ each park, all local government partners were keen to be provided with the intervention. Often local governments’ policies of equitable provision of services to all residents discourage participation in studies with a ‘control arm’ as this is perceived as withholding services/programs (or equivalent). The several follow up time points of evaluation employed in the present study, offer valuable information on a real-world pragmatic public intervention, despite the lack of control parks. A longitudinal study beyond 12 months may be warranted to further evaluate the longer-term impact of this type of public health intervention. Alternatively, a ‘control period’ may also be used as a method to serve as a waiting list ‘control arm’ where outcomes can be compared between a no-intervention period and the intervention period.

Moreover, we had encountered several interruptions and delays outside the control of the research team, including delay in construction work, seasonal weather, COVID-19 restrictions, and variation in park sizes and features. Lastly, we used a validated method of direct observation of park visitors in specific time periods (11 scans), therefore, some visitors, including older people, were excluded from the observations, as they attended the park outside these scan periods. This was evident following our training program where activities/sessions at the park took place in late morning which were not included during the planned scan periods. The complexity of executing such a collaborative project included many confounding factors outside the control of the research team. Finally, the nature of this study including how data were collected regarding park visitors limits how broadly we can generalise our findings, as we were unable to control for potential confounding factors such as age and sex. Despite these limitations, the study design, combining aspects from a natural experiment design enhanced by site activation, offers a unique setting to identify and explore the links between the built environment and visitors’ behaviours which can further influence future park design and its impact on public health.

## Conclusion

Installation of the Seniors Exercise Parks and the supportive programs and activities following six park upgrades resulted in increased older people’s park visitation and engagement in physical activity using the specialised equipment. Installation of age-friendly outdoor equipment alone may not be sufficient to increase older people’s park-based physical activity, highlighting the importance of supporting community engagement and training with ongoing commitment of local governments for sustained impact.

## Data Availability

The datasets generated and/or analysed during the current study are not publicly available due ethical restrictions but are available from the corresponding author on reasonable request.
